# Pathological Buying Online as a Specific Form of Internet Addiction: A Model-Based Experimental Investigation

**DOI:** 10.1371/journal.pone.0140296

**Published:** 2015-10-14

**Authors:** Patrick Trotzke, Katrin Starcke, Astrid Müller, Matthias Brand

**Affiliations:** 1 Department of General Psychology: Cognition, University Duisburg-Essen, Duisburg, Germany; 2 Department of Psychosomatic Medicine and Psychotherapy, Hannover Medical School, Hannover, Germany; 3 Erwin L. Hahn Institute for Magnetic Resonance Imaging, Essen, Germany; Erasmus University Rotterdam, NETHERLANDS

## Abstract

The study aimed to investigate different factors of vulnerability for pathological buying in the online context and to determine whether online pathological buying has parallels to a specific Internet addiction. According to a model of specific Internet addiction by Brand and colleagues, potential vulnerability factors may consist of a predisposing excitability from shopping and as mediating variable, specific Internet use expectancies. Additionally, in line with models on addiction behavior, cue-induced craving should also constitute an important factor for online pathological buying. The theoretical model was tested in this study by investigating 240 female participants with a cue-reactivity paradigm, which was composed of online shopping pictures, to assess excitability from shopping. Craving (before and after the cue-reactivity paradigm) and online shopping expectancies were measured. The tendency for pathological buying and online pathological buying were screened with the Compulsive Buying Scale (CBS) and the Short Internet Addiction Test modified for shopping (s-IATshopping). The results demonstrated that the relationship between individual’s excitability from shopping and online pathological buying tendency was partially mediated by specific Internet use expectancies for online shopping (model’s *R*² = .742, *p* < .001). Furthermore, craving and online pathological buying tendencies were correlated (*r* = .556, *p* < .001), and an increase in craving after the cue presentation was observed solely in individuals scoring high for online pathological buying (*t*(28) = 2.98, *p* < .01, *d* = 0.44). Both screening instruments were correlated (*r* = .517, *p* < .001), and diagnostic concordances as well as divergences were indicated by applying the proposed cut-off criteria. In line with the model for specific Internet addiction, the study identified potential vulnerability factors for online pathological buying and suggests potential parallels. The presence of craving in individuals with a propensity for online pathological buying emphasizes that this behavior merits potential consideration within the non-substance/behavioral addictions.

## Introduction

Pathological buying (PB), compulsive buying, buying addiction, and oniomania are different terminologies describing the same phenomenon in which individuals are preoccupied with shopping, suffer from recurrent buying impulses or episodes, and lose control over their buying behavior [1,2]. This behavioral excess is related to severe negative consequences such as marked distress, social and occupational problems, delinquency, or financial bankruptcy. Estimates of prevalence deriving from studies in the U.S. and Germany range from 5.8 to 8.0% [3–5]. The clinical classification of this phenomenon is still under debate, which is reflected by the different terminologies: Although some authors argue that PB should be classified as impulse control disorder [6,7], others emphasize parallels to an obsessive-compulsive spectrum [2] or to behavioral addictions [8,9]. Therefore, in the current paper, we use the neutral term “pathological buying” in accordance with Müller et al. [10].

With respect to etiology and pathogenesis, different emotional (e.g., pleasure seeking or escape of negative emotions) and cognitive mechanisms (e.g., impulsivity, failure in self-regulation, or decision-making deficits) appear to be involved in the development and maintenance of PB [11–13]. Currently, a growing number of authors emphasize that PB shares several key characteristics with behavioral addictions when comparing the proposed diagnostic criteria for behavioral addictions, which include preoccupation with the behavior, diminished control over the behavior, repeated unsuccessful attempts to cut down or stop the behavior, tolerance, withdrawal, and adverse psychosocial consequences [8,9,14]. More important, studies have also demonstrated cue-reactivity and craving for individuals with PB [15,16]. The parallels between gambling disorder and substance-use disorders, especially with respect to cue-reactivity and craving, led to reclassification of gambling disorder to the new diagnostic category of non-substance addictions within the fifth edition of the Diagnostic and Statistical Manual of Mental Disorders (DSM-5) [17].

Until now, there has been scarce research on PB in the context of the Internet, although in contrast to brick-and-mortar stores, the growth rates of Internet retailing are steadily increasing, which indicates that more and more individuals use online shopping to acquire consumer goods [18,19]. Hence, it is reasonable that problematic buying behaviors now also occur online [20,21]. Previous studies reported that the Internet provides characteristics that seem to encourage PB, such as the opportunity to buy 24 hours a day, to shop from the convenience of the private home, or to use easy payment systems that lead to inadvertent expenses [22–24]. Therefore, the question remains whether online PB is a distinct clinical condition or whether it is the mere translation of PB in conventional retail environments to another medium [22].

In the research field of Internet addiction, Davis [25] was the first to differentiate between a generalized Internet addiction (GIA) and a specific Internet addiction (SIA). GIA is related to a multidimensional overuse of the Internet with a non-specific usage of one application in particular, whereas SIA is characterized by excessive preoccupations and overuse of one specific Internet application [25,26]. It has been postulated that nearly every Internet application can be used in a dysfunctional/addictive manner; the applications used most frequently in a dysfunctional/addictive manner are online gaming and gambling, social network sites, cybersex, and online shopping [27–29].

Recently, Brand et al. [26] refined a model of Internet addiction that interlinks vulnerability factors, cognitions (coping styles, Internet use expectancies), and mechanisms of reinforcement in the context of GIA and SIA. One path within the model explaining the addictive use of one Internet application is the association between a predisposition to receive gratification by the application (as a person’s core characteristic) and the expectancy that the application satisfies specific desires (i.e., Internet use expectancies as a person’s specific cognition). Therefore, the experienced gratification is reinforcing and constitutes one key element in the development and maintenance of the SIA. However, to the best of our knowledge, this relationship has not yet been tested for online PB. Previous studies have described single vulnerability factors in the context of online PB. With respect to Internet use expectancies, different cognitions that especially motivate online PB have been described (such as buying unobserved, avoiding social interaction, the presence of a greater product variety, or the possibility to satisfy an urge to buy more quickly) [23,30]. With respect to a specific predisposition to receive gratification through the Internet application, there is empirical evidence that online PB is associated with higher reward responsiveness, excitement, and enjoyment [31–33]. Given these rewarding features, one potential predisposing factor might constitute cue-reactivity in the context of addiction models [34]. In more detail, it is postulated that in the context of learning mechanisms, the rewarding effect of the drug becomes associated with addiction-related cues (e.g., surroundings, odors, or paraphernalia) leading to incentive salience for these cues [35,36]. This cue-reactivity is often operationalized with subjective ratings (e.g., arousal and urge) as well as physiological responses (e.g., heart rate, skin conductance, or skin temperature) towards addiction-related cues [37,38]. Cue-reactivity differs among individuals and seems suitable as an indicator of excitation from shopping (shopping excitability), which reflects rewarding features in the context of a potential predisposing factor. Transferring the model of Brand et al. [26] to online pathological buying, it is supposed that an individual who has the predisposition to be highly sensitive for excitation from shopping and has the expectation that the use of Internet shopping sites could meet specific needs and goals (e.g., the expectancy to satisfy an urge to buy more quickly online) may use shopping sites more frequently. The experienced gratification reinforces the predisposing shopping excitability as well as the online shopping expectancies, which results in the uncontrolled and addictive use of online shopping [26].

Cue-reactivity (i.e., the excitability from shopping cues) constitutes the emotional/motivational basis for experiencing craving [39,40]. Craving is commonly described as the irresistible desire to consume a substance and is associated with drug seeking and relapse [34]. The cue-reactivity and craving concept has been transferred to behavioral addictions such as gambling, online gaming, or cybersex use [41–44]. Most recently, cue-reactivity and craving were demonstrated in conventional PB in the offline context [15,16]. To the best of our knowledge, cue-reactivity and craving has not been investigated in online PB so far.

**Fig 1:**
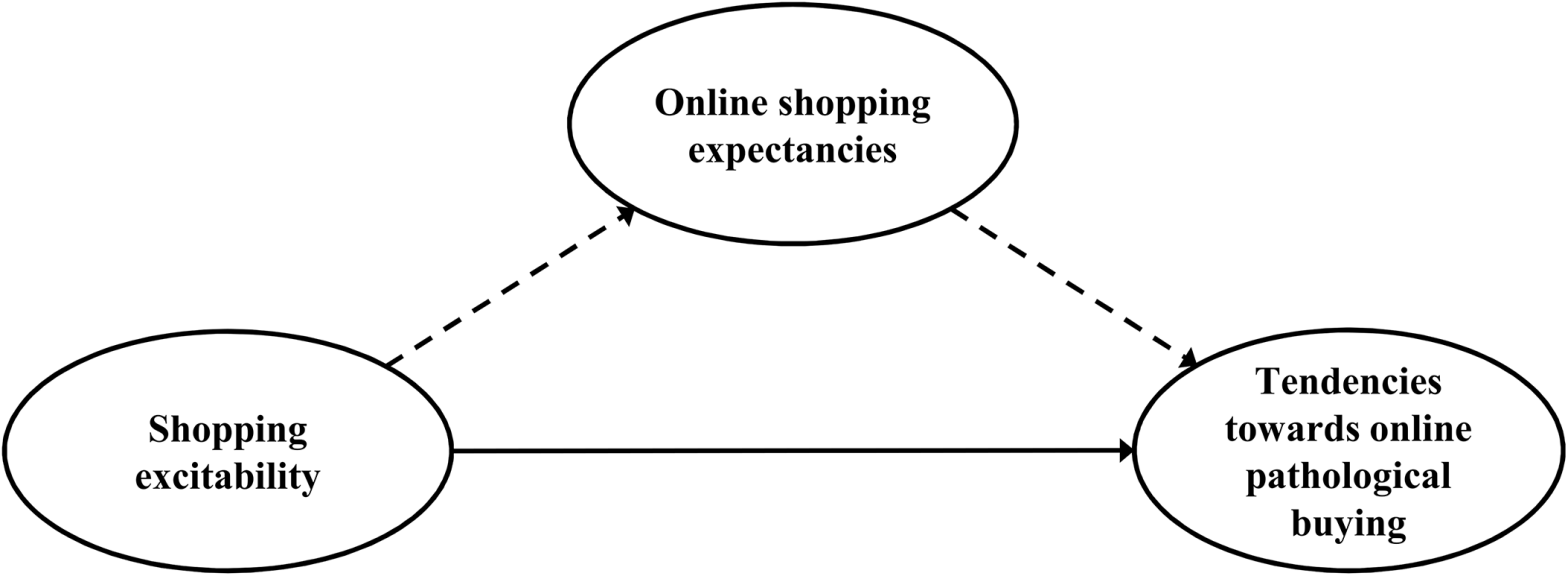
Illustration of the proposed mediation model. The tendencies towards online pathological buying are assumed to be predicted by the predisposing factor shopping excitability (operationalized by variables of cue-reactivity) and online shopping expectancies (operationalized by motives to shop and buy on the Internet). The expectancies should mediate the relationship between shopping excitability and tendencies towards online pathological buying. The direct effect is indicated by the continued arrow; the indirect effects are drawn dashed.

The objectives of the current study were as follows: First, we aimed to determine whether online PB can be conceptualized as SIA because previous research gives reason to assume that online PB is associated with vulnerability factors similar to SIA, such as a higher shopping excitability and specific online shopping expectancies [23,30,32,33,45,46]. In accordance with the proposed SIA model by Brand et al. [26], it is hypothesized that the specific predisposition (shopping excitability) and Internet use expectancies (such as buying anonymously and avoiding social interaction, reaching a greater product variety, and satisfying an urge to buy more quickly) are related to online PB. The higher shopping excitability should not lead exclusively to online PB, but in the context of specific expectancies towards the Internet, online buying will be used with a greater probability, which in turn will lead to addictive use. Transferred to a statistical model, we expect that there is a positive relationship between shopping excitability (operationalized by experimentally induced cue-reactivity) and online PB. We further hypothesized that online shopping expectancies mediate the link between shopping excitability and online PB (Fig 1).

Second, in line with other specific forms of Internet addiction (such as cybersex addiction, gaming, etc.) and conventional PB, we expected a positive relationship between measurements of craving and online PB tendency. Due to the assumed reinforcing effect of buying and the higher shopping excitability in addicted individuals, we hypothesized an increase in craving after exposure to online shopping-related pictures in individuals with a high propensity for online PB and not in individuals with a low propensity.

Third, as outlined before, there is still debate on whether online PB should be considered a distinct clinical condition or a virtual translation of conventional PB. In line with Davis [25], who has argued that in the case of SIA, the pathology could also be developed outside the Internet, we hypothesized that there is a positive relationship between conventional PB and online PB behavior.

## Material and Methods

### Participants

We investigated 240 female participants (mean age *M* = 26.63, *SD* = 10.39, range: 18–64 years; average education duration *M* = 12.34, *SD* = 1.42 years). Only women were included because women were more often represented in clinical samples for PB [1,7,47] and we used online shopping pictures that were selected specifically for women (see section Cue-reactivity paradigm). The participants were recruited via posted flyers at the University Duisburg-Essen and online advertisement in university internal networks. To take part in the study, participants should have bought products online at least once in the last 12 months. The data were collected between January and July 2014. On average, participants spent *M* = 22.93 (*SD* = 19.26) hours online per week, using *M* = 2.84 (*SD* = 3.94) hours for online shopping. All participants took part on a voluntary basis, were not paid, and gave their written informed consent prior to the study. The study was approved by the local ethics committee of the University Duisburg-Essen. The investigation had been conducted according to the principles expressed in the Declaration of Helsinki. The primary sample consisted of 244 female participants, but four participants had to be excluded due to incomplete data sets.

### Instruments

We first evaluated sociodemographic variables to describe the sample in detail; afterwards, we applied the tests described below. All data were collected by trained investigators in a one-to-one setting with Lime Survey (www.limesurvey.org), an open source survey application that was installed on a local server.

#### Short Internet Addiction Test for online shopping

To assess online PB, we used the Short Internet Addiction Test (s-IAT) [48] and modified the items with respect to online shopping (s-IATshopping). The terms “Internet” and “online” were replaced by “Internet shopping sites” or “online shopping activity”. One example is “How often do you try to cut down the amount of time you spend on Internet shopping sites and fail?” This procedure has been used frequently to assess the severity of dependence for specific Internet applications such as online gaming or cybersex [43,49,50]. Twelve items had to be answered on a five point rating scale ranging from 1 (never) to 5 (very often). A sum score was computed ranging from 12 to 60. The s-IAT has good psychometric properties and a sum score > 30 indicates problematic Internet use and a score > 37 a pathological use [48]. The s-IATshopping consists of two subscales: “control/time management” (s-IATshopping I) and “craving/social problems” (s-IATshopping II), which had good internal consistencies in our sample (for the s-IAT I, Cronbach’s α = .85 and for the s-IATshopping II, α = .82).

#### Compulsive buying scale

The compulsive buying scale (CBS) [51] in the validated German version [4] was used to assess a tendency towards PB. It is the most frequently used screener to assess PB in conventional offline retail environments. Seven items had to be answered on five point rating scale ranging from 1 (very often or agreement) to 5 (never or disagreement). A total score was calculated using a regression formula [51]. The original scale ranges from -7.02 to 3.61 and in the validated German version, a cut-off criterion of ≤ -1.09 defines individuals as being at-risk for PB with lower scores in the CBS indicate stronger PB symptoms [4]. In our current study, we inverted the CBS total score in order to ensure that all measurements have positive correlations to get more intuitive values. This means that lower values indicate a lower tendency towards PB symptomatology and higher scores indicate a higher tendency towards PB symptomatology. Hence, the transformed scale ranges from -3.61 to 7.02 and a cut-off criterion of ≥ 1.09 indicates PB. Within the current study, we revealed good internal consistencies (Cronbach’s α = .81).

#### Online shopping expectancies

Inspired by the work of Kukar-Kinney et al. [30], we adopted already existing items and added new items to assess different motives to shop and buy on the Internet. The resulting 20 items had to be answered on a five point rating scale ranging from 1 (absolute disagreement) to 5 (absolute agreement). In a first step, we extracted three factors using Horn’s parallel analysis [52]. In a second step, using exploratory factor analysis (with promax rotation), the items loaded on the following factors: “buying anonymously/avoiding social interaction”, “buying availability/product variety”, and “immediate positive feelings”. In contrast to Kukar-Kinney et al. [30], the factors “buying anonymously” and “avoiding social interaction” yielded one factor due to similarities in regard to content. Furthermore, due to non-loadings, five items were excluded, resulting in a final questionnaire consisting of 15 Items. The items and the results for the factor analysis are presented in S1 Table. The three resulting subscales had good internal consistencies (Cronbach’s α for “buying anonymously/avoiding social interaction”, α = .85, for “buying availability/product variety”, α = .84, and for “immediate positive feelings”, α = .86).

#### Cue-reactivity paradigm

We applied a cue-reactivity paradigm with online shopping pictures to assess shopping excitability, which has been often used in addiction research [53,54]. The paradigm contained distal and proximal shopping pictures because it has been shown that both types of cues elicit craving reactions in individuals suffering from PB [15,55]. The distal shopping pictures were generally related to online shopping and showed cues such as the cover pages of online shopping sites, shopping cart symbols, or payment buttons. The proximal shopping cues contained pictures of specific online shopping products that female pathological buyers usually prefer to buy (i.e., housewares, cosmetics, clothes, shoes, books, jewelries, leatherware, and CD/DVDs; for examples of distal and proximal cues, see S1 Fig) [47,56,57]. The pictures were presented randomly on a screen in a size of 700 x 500 pixels, and the participants had to rate these cues with respect to arousal and urge to buy on a five-point rating scale ranging from 1 (not at all) to 5 (very).

#### Craving assessment

Craving reactions were assessed by using a modified version of the Desires of Alcohol Questionnaire (m-DAQ) [58], which has been modified in recent studies to determine craving reactions towards buying [15,55]. The 14 Items had to be rated on a 7 point rating scale from 0 (absolute disagreement) to 6 (absolute agreement). The mean score was calculated after recoding two inverted items. The questionnaire was administered twice, before and after the presentation of the buying-related pictures within the cue-reactivity paradigm (pre- and post-craving measurement). The internal consistencies for the pre- and post-application were good (Cronbach’s α for the DAQ-pre, α = .85, and for the DAQ-post, α = .88).

### Statistical analyses

With respect to the proposed mediation model, we operationalized the latent variables as follows: The arousal rating and the urge to buy rating of the cue-reactivity paradigm represent the latent dimension “shopping excitability”, whereas the different motives (“buying anonymously/avoiding social interaction”, “buying availability/product variety”, and “immediate positive feelings”) represented the latent dimension “online shopping expectancies”, which represents the hypothesized mediator. The s-IATshopping I (“control/time management”) and the s-IATshopping II (“craving & social problems”) represented the latent dimension “tendencies towards online PB”, which is the proposed dependent variable. All of the requirements for mediation modeling suggested by Baron and Kenny [59] were fulfilled. For the evaluation of the model fit, we applied the standard indices and cut-off criteria [60]: The standardized root mean square residual (SRMR; values below 0.08 indicate good fit with the data), the comparative fit index (CFI), the Tucker Lewis index (TLI; values above 0.90 indicate a good fit, values above 0.95 an excellent fit), and the root mean square error of approximation (RMSEA; “test of close fit”; a value below 0.08 with a significance value below 0.05 indicates acceptable fit). Basic statistical analyses were performed using SPSS 22 (IBM Corp., Armonk, USA) and structural equation modeling was performed using MPlus 6.0 [61]. For the bivariate correlational analysis, we applied Pearson’s correlations. With respect to the craving reactions, we divided the sample and compared the participants who scored one standard deviation below and one standard deviation above the mean score of the s-IATshopping (see section Craving reactions within the results). The craving reactions were analyzed using analysis of variance (ANOVA), with the between-subject factor “group” (scoring low vs. scoring high for online PB) and the within-subject factor “time” (pre- vs. post-craving measurement). The effect sizes were determined by using partial η² (η_p_²). Post-hoc *t*-tests were performed, and Cohen’s *d* [62] was calculated to indicate effect sizes. To evaluate diagnostic concordances and divergences, the described cut-off criteria (see section Instruments) were used for the CBS and the s-IAT-shopping to describe the number of classified participants and calculate the interrater reliability (Cohen’s Kappa; κ).

## Results

A description of the variables used in the questionnaires and the experimental paradigm are shown in Table 1.

**Table 1:** Description of the questionnaires and the experimental paradigm. s-IATshopping = Short Internet Addiction Test modified for shopping; CBS = Compulsive Buying Scale

*N* = 240	*M*	*SD*
**s-IATshopping**	18.88	6.24
**s-IATshopping I**	10.96	4.03
**s-IATshopping II**	7.92	2.73
**CBS-Score** ^a^	-1.03	1.83
**Arousal**	2.46	0.77
**Urge to buy**	2.41	0.82
**Anonym**	1.71	0.79
**Variety**	3.48	0.91
**Immediate positive feelings**	1.79	0.86

^a^ CBS-score transformed; lower levels indicate lower levels of pathological buying.

### Shopping excitability and online shopping expectancies

**Fig 2:**
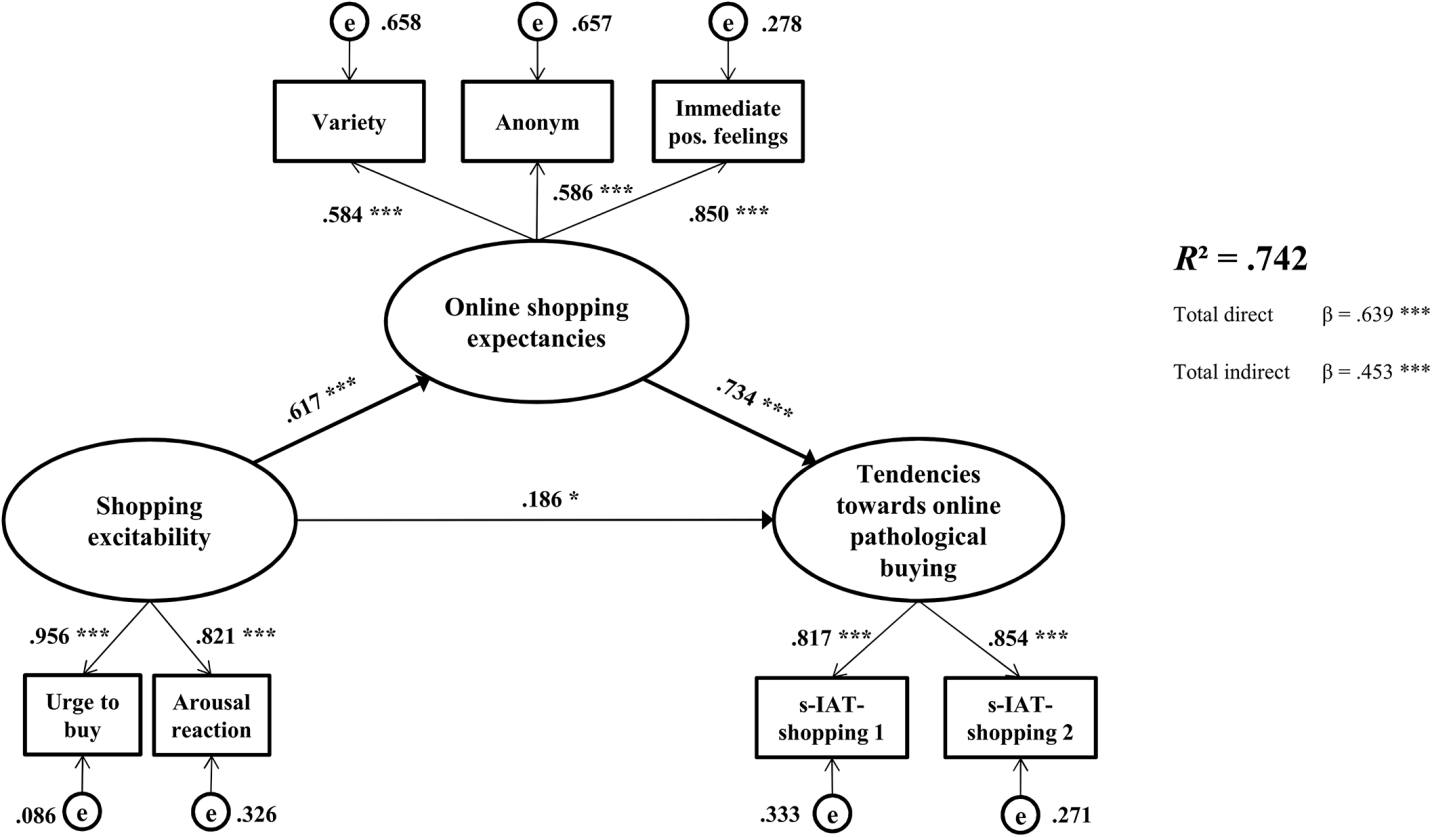
Results for the structural equation model. The factor loadings of the latent dimensions and the β-weights with the significance, as well as the total direct and total indirect effects, are depicted. e = error *** *p* < .001, ** *p* < .01, * *p* < .05

For the mediation model, the relevant variables were entered into the mediation model in accordance with the hypotheses. The effect sizes of the correlations between the manifest variables are moderate to high and are presented in Table 2. The model fits well with the data (CFI = .97; TLI = .95; RMSEA = .09, *p* < .05; SRMR = .034), although the RMSEA was somewhat high. For the whole model, the χ²-test was significant (χ² = 33.76, *df* = 11, *p* < .001), but the χ²/*df* ratio was next to 3. Overall, 74.2% of the variance within online PB was explained (*R*² = .742, *p* < .001). The direct and indirect effects are shown in Fig 2.

**Table 2:** Correlations of the manifest variables of the mediation model. The manifest variables “Urge to buy” and “Arousal” were assessed experimentally using the cue-reactivity paradigm, whereas the variables “Variety”, “Anonym”, and “Immediate positive feelings” were assessed by questionnaire. s-IATshopping = Short Internet Addiction Test modified for shopping

	s-IAT shopping	Urge to buy	Arousal	Variety	Anonym	Immediate positive feelings
**Urge to buy**	.547***					
**Arousal**	.483***	.785***				
**Variety**	.453***	.378***	.370***			
**Anonym**	.487***	.312***	.255***	.247***		
**Immediate positive feelings**	.636***	.504***	.411***	.524***	.499***	

*** *p* < .001

The direct effect of the predictor “shopping excitability” on the criterion “tendencies towards online PB” was significant (β = .186, *SE* = .08, *p* < .05). The direct effect from the predictor “shopping excitability” to the mediator “online shopping expectancies” was also significant (β = .617, *SE* = .05, *p* < .001), as well as the effect from the mediator “online shopping expectancies” to the criterion “tendencies towards online PB” (β = .734, *SE* = .08, *p* > .001). Furthermore, the total indirect effect from “shopping excitability” over “online shopping expectancies” to “tendencies towards online PB” was significant (β = .453, *SE* = .07, *p* < .001). Given that the direct effect remained significant after including the mediator, a partial mediation was observed.

### Craving reactions

**Fig 3:**
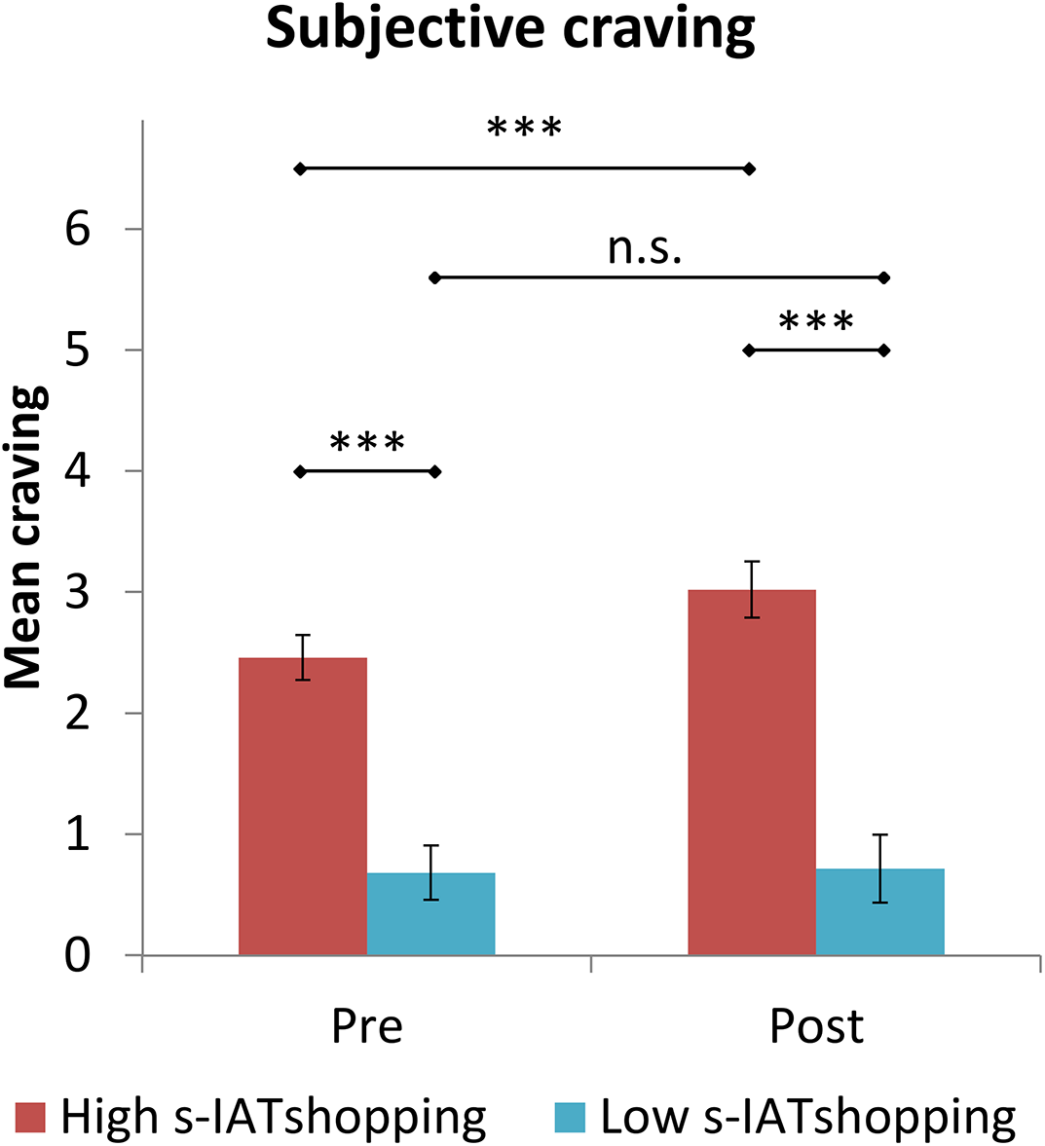
Subjective craving reactions. Results with respect to the craving reactions pre- and post-cue presentation for the participants scoring high and low on the s-IAT-shopping. Error bars represent standard deviation (SD). *** *p* < .001, n.s. = not significant.

There were high correlations between the s-IATshopping and the craving before the online shopping picture presentation (pre-carving; *r* = .556, *p* < .001), as well as after the picture presentation (post-craving; *r* = .580, *p* < .001). To demonstrate that the increase in craving was solely observed in the individuals scoring high for online PB, we divided the sample and compared the participants who scored one standard deviation below and one standard deviation above the mean score of the s-IATshopping. A total of *n* = 49 participants scored at the upper and lower limits of the distribution. To compare the means of the respective craving scores, we used ANOVA with the between-subject factor “group” (scoring low vs. scoring high for online PB) and the within-subject factor “time” (pre- vs. post-craving measurement). We observed a significant main effect for “group”, *F*(1, 47) = 43.99, *p* < .001, η_*p*_² = 0.48; and a significant main effect for “time”, *F*(1, 47) = 6.09, *p* < .01, η_*p*_² = .12. The significant “group × time” interaction, *F*(1, 47) = 4.80, *p* < .05, η_*p*_² = .09, indicated that the participants scoring high or low for online PB reacted differently across the two administration times. Post-hoc *t*-tests revealed an increase in craving after the picture presentation, *t*(28) = 2.98; *p* < .01, *d* = 0.44 for the individuals scoring high for online PB, whereas no changes were observed for individuals scoring low, *t*(28) = .35, *p* = .73, *d* = 0.04. In addition, the participants scoring high for online PB showed higher cravings before (*t*(47) = 6.08, *p* < .001, *d* = 1.81) and after (*t*(47) = 6.32, *p* < .001, *d* = 1.88) the picture presentation in comparison with the individuals scoring low for online PB (Fig 3).

### Clinical screening instruments

**Fig 4:**
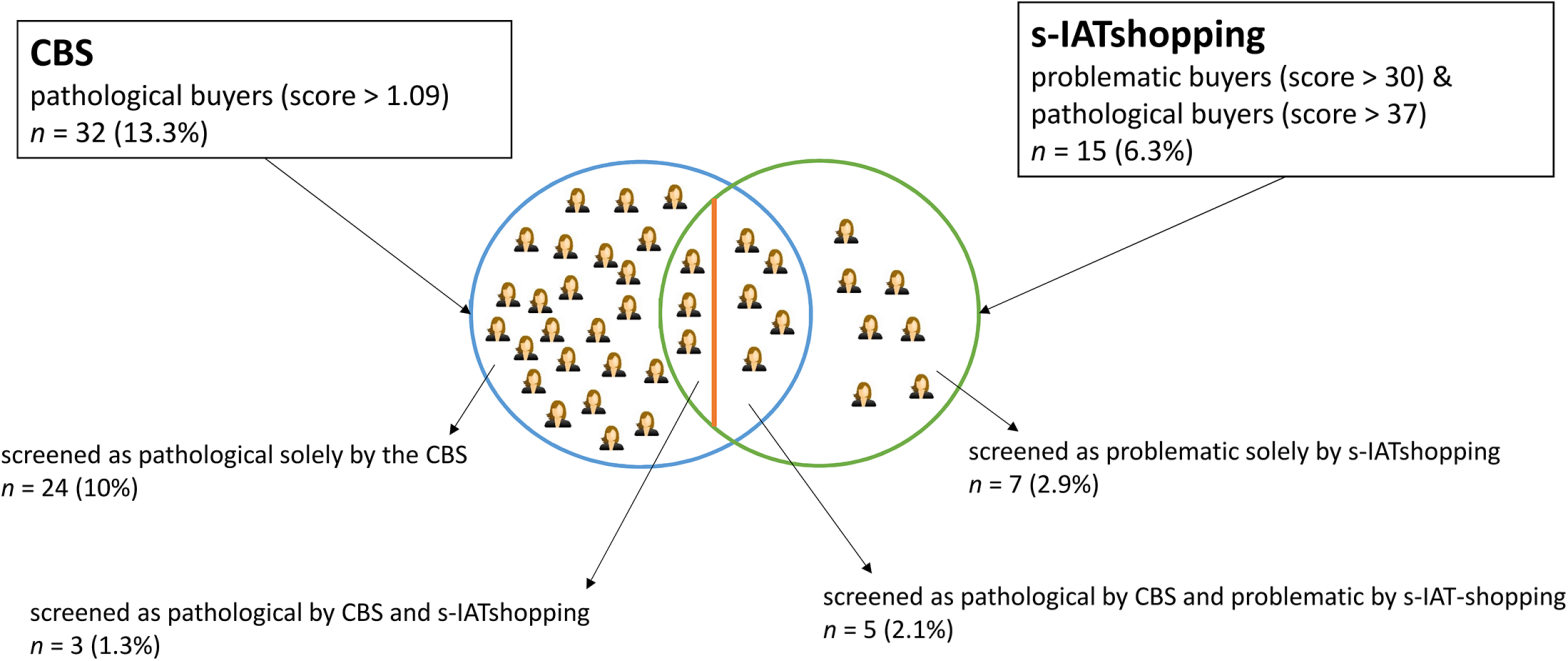
Frequencies of participants with problematic buying behavior. Illustration of the frequencies of those participants who were screened as pathological buyers by the Compulsive Buying Scale (CBS; total score > 1.09) and as problematic/pathological buyers by the Short Internet Addiction Test modified for shopping (s-IATshopping; problematic score > 30; pathological score > 37). Whole sample size *N* = 240.

There was a strong correlation between the s-IATshopping and the CBS, *r* = .517, *p* < .001 (note that CBS total scores were inverted). Fig 4 depicts the frequencies of individuals detected as having PB (screened by the CBS with total scores > 1.09) and problematic as well as pathological use in the online context (screened by the s-IATshopping with problematic scores > 30 and pathological scores > 37). By applying the described cut-off criteria, some of the individuals within our samples were classified as pathological buyers solely by the CBS (*n* = 24), whereas some were classified as having problematic online buying behavior solely by the s-IATshopping (*n* = 7). In addition, there was an overlap of *n* = 8 participants (3.3%) fulfilling the criteria for pathological buying behavior as assessed by the CBS and the s-IAT-shopping. This overlap is evaluated as only mild with κ = .278, *p* < .001.

## Discussion

This study investigated whether online PB can be conceptualized as a SIA by investigating vulnerability factors for online PB, such as shopping excitability, specific Internet use expectancies and craving. Furthermore, the relationship of online PB and conventional PB measurements was investigated to explore whether online PB can be conceptualized as being independent from conventional PB. The results demonstrated, in line with the proposed model for SIA by Brand et al. [26], that the relationship of shopping excitability and the tendency towards online PB was partially mediated by the online shopping expectancies. Furthermore, craving reactions were correlated with online PB, and an increase in craving was observed after picture exposure only in those individuals who scored high for online PB. In addition, it was demonstrated that the two screening instruments (CBS and s-IATshopping) were correlated moderately, but there were differences and overlaps with respect to the diagnostic properties when applying the proposed cut-off scores. The results should be discussed in the context of the “Internet addiction” concept and with respect to mechanisms potentially contributing to the development and maintenance of addictive disorders.

Indicators of shopping excitability predicted online PB tendencies. One strength of the study is that shopping excitability was operationalized experimentally using variables of cue-reactivity (arousal and urge to buy) instead of questionnaire self-reports. Previous research demonstrated that drug-relevant cue exposure activated a generalized motivational state (including arousal), which is strongly related to reward approach behavior in addicted individuals [34,36]. Anticipating and receiving reward has been noted to be important in the development and maintenance of substance and non-substance-related addictions such as gambling disorder, pathological online gaming, or pathological cybersex use [41,44,63]. The rewarding and exciting nature of buying was demonstrated in previous studies in the conventional [7] and in the online context [31,32,64]. Therefore, the act of buying was described as being used to modulate mood and provide relief or escape from negative emotions [57,65]. Given these parallels, we argue for the addictive nature of buying in the online context.

The higher sensitivity for excitation from shopping does not lead exclusively to the buying pathology. Further influencing factors include expectations that buying on the Internet meets specific needs and goals. In accordance with Kukar-Kinney et al. [30], we explored three different expectancies that motivate especially online buying in contrast to conventional brick-and-mortar store buying. These expectancies were as follows: (1) buying anonymously and avoiding social interaction, (2) buying availability and reaching a greater product variety, and (3) receiving immediate positive feelings. Previous research showed that individuals with PB feel shame and regret after their buying episodes, so it is plausible that they may not want others (especially family members) to see what, how much, and how often they buy [10,30]. In addition, anxiety (especially social anxiety) is often associated with PB [66]. Consequently, the anonymity and the non-existent social interaction within the online shopping environment may intensify the preference for buying online [30]. A greater variety of products and brands and the perpetual availability provide a way for pathological buyers to achieve greater and more direct positive feelings [30], and it is proposed that the Internet with its ease, availability and convenience should satisfy needs and desires more instantly in contrast to conventional shopping [22,30]. Previous studies already emphasized the importance of specific expectancies in association with online PB [23,24,64]. Consistent with our results, Dittmar et al. [24] demonstrated that emotional and identity-related expectancies mediate the relationship between a predisposing factor (materialistic value orientation) and online PB tendencies.

Altogether, there is empirical evidence that the phenomenon of online PB is associated with specific factors of vulnerability (a predisposing shopping excitability and online shopping use expectancies), and these factors are interlinked as proposed in the currently published model for SIA by Brand et al. [26]. The model revealed that online PB is not singularly explained by specific predispositions such as excitability from shopping. The results demonstrated that Internet use expectancies were associated with online PB and partially mediated the relationship between shopping excitability and online PB tendencies, which increases the probability of using online shopping sites excessively. It is assumed that individuals with a high sensitivity for excitability from shopping who have the expectancy that online shopping meets specific needs and goals should be more prone to gratification from online buying and should therefore be more at risk for developing online PB. This means that it is suggested that a general excitability is not enough to develop online PB. Only if individuals also have the expectancy that their desires can be satisfied by online shopping (which is impacted by the predisposing factors), they use such Internet applications and experience gratification, which in turn reinforces the expectancies. This mediation model explained a great proportion of the variance (74.2%) within the tendency towards online PB measured by the s-IATshopping. More importantly, the predisposition for excitability from shopping determines why individuals develop the expectancy that using online shopping sites can satisfy their desires and then use online shopping sites addictively and not, for example, cybersex sites. The study, although preliminary, suggests parallels between online PB and SIA. This is the first study that tries to conceptualize the phenomenology of online PB in the context of Internet addiction, and the interpretations should be taken with care. However, this attempt has the advantage that it provides a theoretical base for testing hypotheses to confirm or refute conceptualizations of Internet addiction.

In addiction theory, it is postulated that cue-reactivity and craving have been fundamental mechanisms within the development and maintenance of addictions [36]. Cue-reactivity represents the manner in which addiction-related cues become associated with the reinforcing mechanisms of the drug and has been used to operationalize excitability from shopping (i.e., arousal and urge to buy ratings) [35,39]. However, cue-induced sensitization of incentive motivational processes and brain functions (especially the mesolimbic dopaminergic system) provide the emotional/motivational basis for experiencing craving [36,40,67,68]. Our results demonstrated that individuals who score high for online PB show higher cravings (assessed by the questionnaire) before and after cue exposure–in contrast to individuals scoring low, who were non-reactive. These findings coincide with studies showing cue-reactivity and craving reactions for PB patients in conventional buying environments [15,16]. Previous research demonstrated similarities between individuals with substance addictions and behavioral addictions (i.e., gambling disorder) with respect to cue-induced craving on subjective and brain functional levels [69–71]. In consequence, within the DSM-5, the concept of substance abuse and dependency has been extended to the new diagnostic category of non-substance-related addictions [17]. Until now, only gambling disorder has been included, but it has been mentioned that Internet gaming disorder is one condition with a current need for further research [17]. It has already been argued that the addictive use of the Internet can also focus on other applications such as cybersex, shopping, or social network sites [26,29]. To the best of our knowledge, this is the first study demonstrating cue-induced craving in online PB. Given the parallels to other behavioral addictions and to the concept of Internet addiction, we suggest a potential consideration of online PB in the context of SIA within the category of behavioral/non-substance-related addictions. As outlined before, these are preliminary results, and the empirical basis for these considerations is not sufficiently evaluated; more research is needed to better understand the phenomenology and clinical aspects of the pathological behavior.

With respect to the question of whether online PB is only “an old problem in a new marketplace” ([22] p. 739), the results demonstrated that the s-IATshopping and the CBS were moderately correlated. Previous studies reported heterogeneous findings, and PB was sometimes related to a dysfunctional use of the Internet [64,72], and sometimes not [24,32]. However, most studies published in the context of online PB have been conducted without differentiating between GIA and SIA. Therefore, it is probable that in the case of non-correlation, the applied questionnaires reflect instead a general dysfunctional use of the Internet, for instance, an addictive use without a first-choice application (conceptualized as GIA). Online PB has characteristics of a SIA (as shown by the mediation model), and it seems that the phenomenon has to be screened specifically (e.g., using the s-IAT shopping instead of the original IAT or comparable scales). This circumstance indicates again parallels between online PB and SIA. In line with this assumption, by applying the proposed cut-off criteria for the screening instruments (CBS & s-IATshopping), there were individuals who were screened as pathological buyers solely either by the s-IATshopping or by the CBS, which indicated that there seemed to be specific features in the online context that could only be detected by specific screening instruments. This argument is supported by the study of LaRose and Eastin [64], who demonstrated that Internet domain-specific measurements were related to unregulated online buying, whereas offline measures were not. However, our findings also demonstrated that there are diagnostic concordances indicating that some individuals with pathological tendencies could be classified by both instruments. We interpret these findings, in line with Davis [25], as implying that PB could also be developed outside the Internet and that the online environment with its specific features (such as advertising pop-ups, timed discount offers, and vivid graphical displays) could aggravate the pathology.

The parallels between online PB and specific Internet addiction provide some clinical implications that should be sketched briefly. There is evidence that within the therapeutic context, cognitive-behavioral therapy (CBT) is the method of choice for the treatment of both PB [73,74] and Internet addiction [75,76]. With respect to our results, specific predispositions and cognitions seem to be related to the problematic behavior. Consequently, as proposed in the review by Brand et al. [26], self-monitoring techniques could be used to determine situational, emotional, and cognitive conditions as well as patterns of positive and negative reinforcement associated to PB. Furthermore, cognitive restructuring and reframing could be applied to change the described Internet use expectancies and negative feelings could be applied to establish new healthy buying patterns. Due to cue-reactivity for individuals with a high tendency for online PB, future therapeutic trials should explore the efficacy of cue-exposure or stimulus control techniques in the treatment context.

The current findings should also be discussed in light of some limitations. The main proportion of the sample was composed of female students, which limits the generalizability to the female German population. Another limiting factor may derive from the circumstance that income and ethnicity were not assessed. However, the goal of the study was to examine online PB, and the student sample seems suitable because it has often been reported that younger people use the Internet more frequently for shopping and younger age is an important predictor for PB [3–5]. Taking into account that students in Germany have good, interest-free credit accounts and the assumption that PB is not linked to education, the sample is comparable with young female adults in full-time employment.

Within our non-clinical sample, we did not control for potential influencing emotional factors (such as depression or anxiety) and did not assess confounding psychiatric comorbid conditions (e.g., obsessive-compulsive disorder or hoarding disorder). Future studies should control for potential influencing factors such as depression, anxiety and comorbid conditions to improve the specificity of the results with respect to addictive disorders. With respect to female samples, future studies should assess the menstrual cycle because it is known to influence craving for other behaviors such as smoking and eating [77,78].

Another limitation is that online shopping expectancies were measured by a newly developed questionnaire that had not been tested previously. Although the validity is not completely evaluated, the item development was inspired by a previous work of Kukar-Kinney et al. [30], and we revealed a similar factor structure using exploratory factor analysis with good internal consistencies. Furthermore, there is no valid scale to screen online PB. Due to this circumstance, we used the s-IAT in a modified version for shopping and applied the cut-off criteria, which derived from investigations towards Internet addiction in a German sample. This procedure had often been applied in studies investigating the pathological use of specific online activities such as gaming, or cybersex [42,49].

Future studies should investigate further vulnerability and cognitive factors potentially related to online PB (e.g., stress vulnerability, psychopathologies, or coping styles). Likewise, it would make sense to investigate male participants because population-based surveys indicated that there were no gender differences with respect to the prevalence of PB [3,4]. Within this first study, cue-reactivity and craving were investigated by subjective valuations, so future aims should focus on the assessment of peripheral physiological responses (e.g., electrodermal activity, skin temperature, or heart rate) and brain activity using functional techniques like previous studies on Internet addiction [44,79]. Finally, the presented model should be tested in clinical samples with patients suffering from online PB.

## Conclusion

The study provided the first indications of potential vulnerability factors for online PB (i.e., excitability from shopping and specific Internet use expectancies), which suggests parallels to SIA. Given the parallels between online PB and other addictions with regard to craving, we argue for a potential consideration of online PB within the diagnostic category of non-substance/behavioral addictions. The applied screening instruments supported this view and led to the assumption that there are distinct features in the online context requiring specific screening instruments for online PB.

## Supporting Information

S1 Fig Example for the stimuli of the cue-reactivity paradigm. (A) distal online shopping cue (content of a shopping cart); and (B, C) proximal online shopping cues (clothes, cosmetics).
(PPTX)

S1 Table Factor loadings and means of the items of the Internet shopping use expectancies questionnaire. (DOCX)
