# Supplementary figures and images for: Pathological Buying Online as a Specific Form of Internet Addiction: A Model-Based Experimental Investigation
Source: PLoS One. 2015 Oct 14;10(10):e0140296. doi: 10.1371/journal.pone.0140296 (PMC4605699; doi:10.1371/journal.pone.0140296)

## Slide 1
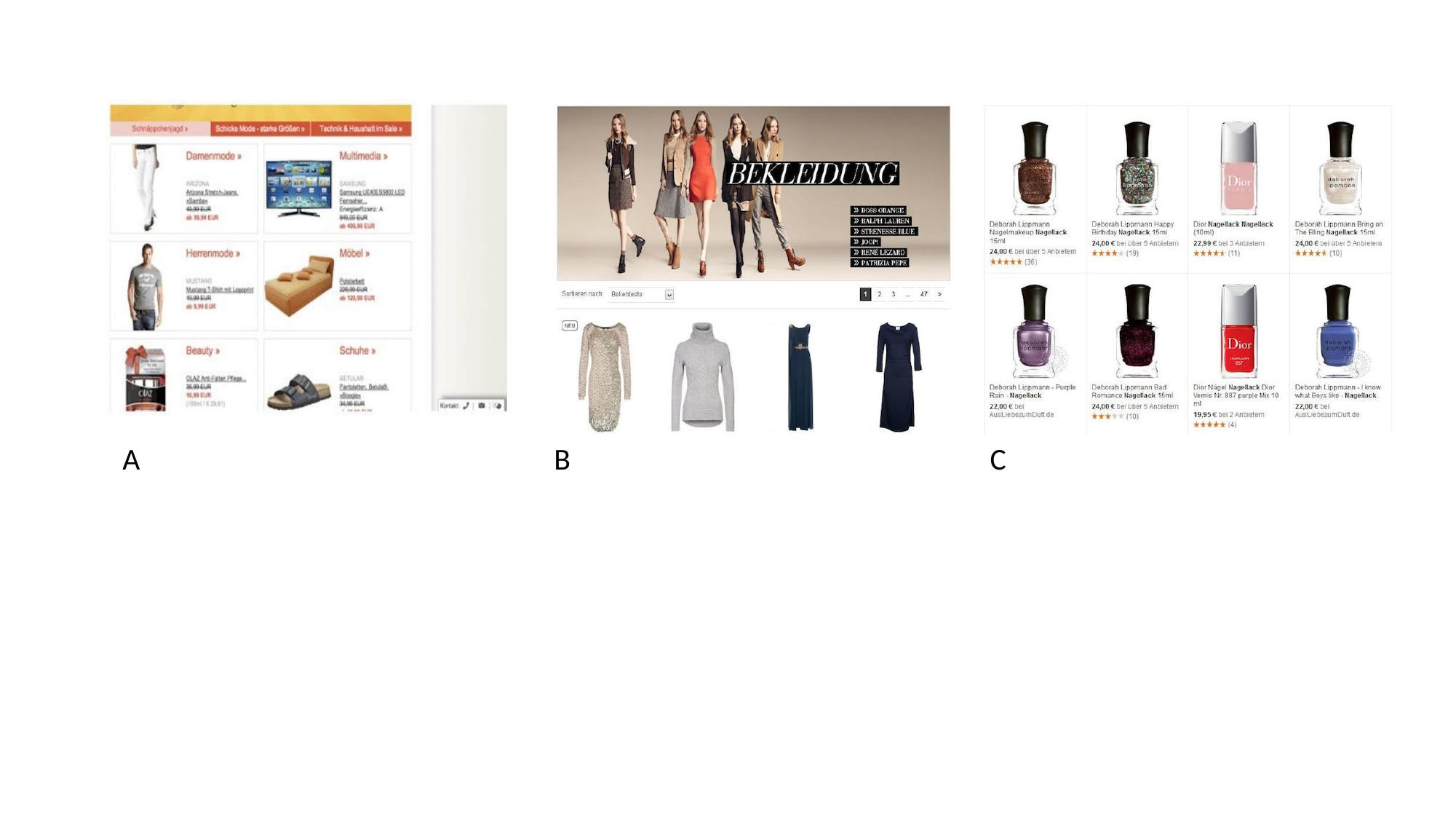

A
B
C

Supplement: S1 Fig — (A) distal online shopping cue (content of a shopping cart); and (B, C) proximal online shopping cues (clothes, cosmetics). (PPTX) [file pone.0140296.s001.pptx]
