# Supplementary material for: Pathological Buying Online as a Specific Form of Internet Addiction: A Model-Based Experimental Investigation
Source: PLoS One. 2015 Oct 14;10(10):e0140296. doi: 10.1371/journal.pone.0140296 (PMC4605699; doi:10.1371/journal.pone.0140296)
Supplement: S1 Table — (DOCX) [file pone.0140296.s002.docx]

**S1 Table. Factor loadings and means of the items of the Internet shopping use expectancies questionnaire.**

| **Item no.** | **Item**  **“I prefer buying on the Internet because…”** | **Factor** | | | **M** | **SD** |
| --- | --- | --- | --- | --- | --- | --- |
|  |  | **1** | **2** | **3** |  |  |
| **1** | **… no one can see me buy.** | **.858** | -.008 | -.055 | 1.70 | 1.015 |
| **14** | **… no one can see what I buy.** | **.782** | -.100 | .129 | 1.62 | 1.016 |
| **2** | **… I can avoid other shoppers.** | **.701** | .201 | -.184 | 1.76 | 1.018 |
| **7** | **… I do not like the interaction with the sales clerks.** | **.682** | .091 | -.013 | 1.86 | 1.033 |
| **8** | **… I do not like to shop where people know me.** | **.655** | -.043 | .010 | 1.58 | 0.902 |
| **5** | … no one can see how much I buy. | **.528** | -.098 | .340 | 1.47 | 0.891 |
| **18** | … I do not have to feel ashamed online if I do not buy something. | **.473** | -.036 | .166 | 1.56 | 0.979 |
| **10** | **… I like to see a lot of products in a short time.** | -.104 | **.771** | .084 | 3.58 | 1.158 |
| **9** | **… there are more choices of where to shop.** | .022 | **.765** | -.060 | 3.82 | 1.112 |
| **3** | **… there are more product choices.** | .084 | **.739** | -.035 | 3.89 | 1.059 |
| **16** | **… I can gather a lot of information on the latest fashions in a short time.** | -.133 | **.666** | .185 | 3.21 | 1.274 |
| **17** | **… I can buy 24 hours a day.** | .023 | **.576** | .135 | 3.53 | 1.287 |
| **15** | **… it is easier to find things I like available in the right size.** | -.093 | **.512** | .075 | 2.84 | 1.402 |
| **20** | … I do not mind waiting for the merchandise to be delivered. | .218 | **.497** | -.136 | 2.67 | 1.184 |
| **19** | … I do not miss the atmosphere in conventional shopping environments. | .380 | **.383** | -.079 | 2.20 | 1.168 |
| **12** | **… Internet buying can get me out of a bad mood faster.** | .073 | -.008 | **.780** | 1.75 | 1.009 |
| **11** | **… I can get more immediate pleasure from buying.** | -.029 | .073 | **.768** | 1.84 | 1.006 |
| **4** | **… I can satisfy my urge to shop and buy faster.** | -.058 | .266 | **.638** | 1.95 | 1.162 |
| **13** | **… the Internet shopping experience is more exciting.** | .174 | .149 | **.543** | 1.65 | 0.916 |
| **6** | … it is easier to shop without my family or partner knowing. | .381 | -.212 | **.446** | 1.58 | 1.011 |

***Note:*** only the bold-faced items were included for further analysis.
